# Cognitive effects on experienced duration and speed of time, prospectively, retrospectively, in and out of lockdown

**DOI:** 10.1038/s41598-023-50752-7

**Published:** 2024-01-23

**Authors:** Cyril Nicolaï, Maximilien Chaumon, Virginie van Wassenhove

**Affiliations:** 1 NeuroSpin, Cognitive Neuroimaging Unit, CEA, INSERM, Université Paris-Saclay, 91191 Gif/Yvette, France; 2 École Normale Supérieure, PSL, 75005 Paris, France; 3 Institut du Cerveau, ICM, INSERM, CNRS, Sorbonne Université, APHP, CENIR, 75013 Paris, France

**Keywords:** Attention, Perception

## Abstract

Psychological time is influenced by multiple factors such as arousal, emotion, attention and memory. While laboratory observations are well documented, it remains unclear whether cognitive effects on time perception replicate in real-life settings. This study exploits a set of data collected online during the Covid-19 pandemic, where participants completed a verbal working memory (WM) task in which their cognitive load was manipulated using a parametric n-back (1-back, 3-back). At the end of every WM trial, participants estimated the duration of that trial and rated the speed at which they perceived time was passing. In this within-participant design, we initially tested whether the amount of information stored in WM affected time perception in opposite directions depending on whether duration was estimated prospectively (i.e., when participants attend to time) or retrospectively (i.e., when participants do not attend to time). Second, we tested the same working hypothesis for the felt passage of time, which may capture a distinct phenomenology. Third, we examined the link between duration and speed of time, and found that short durations tended to be perceived as fast. Last, we contrasted two groups of individuals tested in and out of lockdown to evaluate the impact of social isolation. We show that duration and speed estimations were differentially affected by social isolation. We discuss and conclude on the influence of cognitive load on various experiences of time.

## Introduction

Time perception is notoriously labile, influenced by numerous factors ranging from basic physiological states such as arousal to cognitive factors such as one’s temporal perspective, emotion, or cognitive load. Time is so fundamental to human life that an altered sense of it is often an indicator of an individual's well-being^1^. During the Covid-19 pandemic, a consortium of researchers collected a series of online tests and questionnaires in several countries, in and out of lockdown, to preserve a quantitative historical record of subjective temporalities and potential covariates^2^. Using the *Blursday* database, the present study asks whether time perception is parametrically altered when participants’ task load is varied during the time interval to be estimated. We assessed time perception using four different behavioral measures: prospective and retrospective duration estimation, and prospective and retrospective experienced speed of the passage of time.

Prospective and retrospective duration estimation consist of asking participants to provide an estimate of a time interval that has just elapsed^3,4^. In a prospective task, participants know beforehand that they have to estimate or classify an elapsed time as a duration: participants’ attention is likely oriented towards time^5^. In a retrospective task, participants are not informed beforehand that they will have to estimate a duration; their attention is a priori not oriented towards time. Retrospective durations are tested with a single trial to prevent participants’ subsequent attentional re-orientation to time. Due to this important experimental limitation, retrospective duration has been less studied^6^ and can benefit from studies with large sample sizes^2,7^.

Whether prospective and retrospective duration estimation share common or distinct mechanisms is a matter of debate. Proponents of shared resources between retrospective and prospective tasks (Brown, 1985; Brown & Stubbs, 1988) have compared and discussed some of their inconsistencies, whereas others (Block & Zakay, 1996; Zakay & Block, 1997) have suggested that while prospective timing may use attentional resources, retrospective timing likely uses memory resources (e.g. Hicks, 1992). Accordingly, prospective and retrospective duration estimates have sometimes been referred to as experienced and remembered durations, respectively^8^.

In their influential review, Block et al. (2010) provided a taxonomy of the cognitive factors that could differentially influence prospective and retrospective duration estimation. These factors included attentional and response demands, task familiarity, task difficulty, memory load, and interindividual variability in working memory (WM) capacity. Their meta-analysis provided key insights on the opposite effects of cognitive load on prospective and retrospective duration: when participants estimate a duration prospectively, an increase in cognitive load decreases the subjective-to-objective duration ratio; conversely, when participants estimate a duration retrospectively, an increase in cognitive load increases the subjective-to-objective duration ratio. The authors concluded that participants’ subjective duration is a good indicator of their experienced cognitive load.

Consistent with this, duration estimation is typically viewed as an information-theoretic measure, whose outcomes result from some form of counting mechanism^9–16^ influenced by processing demands^4,17^. Recent statistical modeling approaches have shown that duration estimation can effectively result from cumulative perceptual changes^18,19^ as well as changes in expectations^20–22^, both likely contributing to perceived speed of time-in-passing.

However, if duration and “time-in-passing” (now referred to as “passage of time”) have often been used interchangeably^17^, it has also been argued that passage of time judgments are distinct from duration estimates and better capture the subjective phenomenology of experiencing time passing in real life^23–25^. Experienced speed of the passage of time judgments (PoT) consist of asking participants to rate how quickly they felt time is passing, using a Likert or a visual-analog scale. The distinctiveness of PoT from duration estimation has been observed for durations in the order of minutes^26^. Whether PoT are covariates or radically different from typical duration estimations and temporal expectation quantifications remains largely unknown, although a recent study using virtual reality suggests that duration and PoT may capture different cognitive processes^27^.

In the present study, we tested a series of working hypotheses that replicate and largely extend previous literature. Using the *Blursday* database^2^, we first sought to replicate the observation that parametrically increasing task load shortens participants’ estimates of elapsed time during the task^28^. In the original experimental design of Polti et al. (2018), task load was manipulated using an n-back WM task. The motivation for using an n-back WM task to interfere with temporal processes is manifold. First, the n-back task uses verbal memory, which prevents participants from overtly counting to estimate elapsed duration^29^. Second, this interference is specific and involves updating processes because the number of items (n) must be maintained in an active state in working memory along with the temporal identity or ordinality of the item^30^. Third, the updating processes in executive functions have been specifically linked to interference effects in duration estimation^31^. Last, they also involve brain structures known to play an important role in timing, such as the striatum and dorsolateral prefrontal cortex, but also the parietal cortex and Broca’s region^30,32–35^.

**Figure 1:**
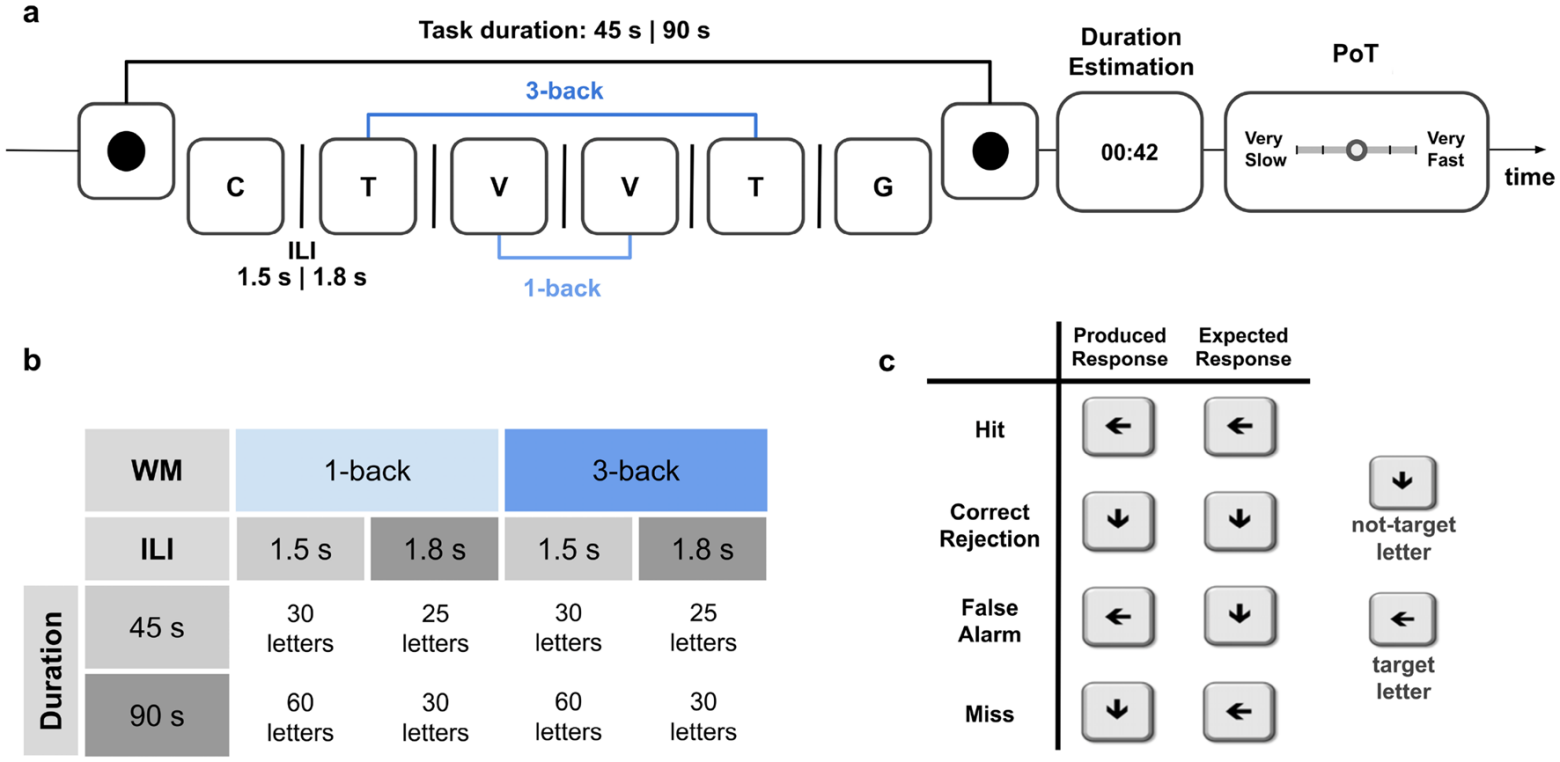
Experimental design. (**a**) A trial of the n-back WM task started with the presentation of a dot on the screen followed by the task instructions. The sequence of letters was presented sequentially and participants performed a 1-back (light blue) or a 3-back (dark blue) WM task. A sequence could last 45 s or 90 s. The inter-letter-interval (ILI) could be 1.5 s or 1.8 s. The WM trial ended with the presentation of a dot. Following the n-back trial, participants were prompted with the duration estimation asking them to estimate the elapsed time between the two dots in minutes: seconds (e.g., 00:42). After the duration estimation, participants were asked to rate their experienced speed of passage of time (PoT) using a five steps Likert scale ranging from “Very Slow” to “Very Fast”. (**b**) The within-participant factorial design WM task (2: 1-back, 3-back) x Duration (2: 45 s, 90 s) x ILI (2: 1.5 s, 1.8 s) is presented in panel b. (**c**) Participants provided an answer after each letter using the leftward arrow for “same” or the downward arrow for “different”. A forced response after each letter in the sequence allowed to quantify the full range of possible responses (Hits, Correct Rejections, Misses, and False Alarms) and assessing WM performance using Signal Detection Theory.

We designed the n-back WM task (Fig. 1) to use signal detection theory and assess participants’ behavioral performance by quantifying their sensitivity ($d^{\prime }$) and bias ($\beta$)^36^. This approach allowed us to assess how the task load, but also participants’ sensitivity and bias, affected retrospective judgments (single-task mode) and the concurrent temporal estimates in the prospective judgments (dual-task mode). After each n-back sequence, participants were required to make a duration and speed estimate of the just elapsed interval while performing the n-back task. Participants engaged in the first n-back sequence were unaware that subsequent timing judgments would be required: thus, the first sequence in the study provided a measure of retrospective duration and PoT. Subsequent trials provided data for prospective duration and PoT.

This approach provides a unique 2 × 2 within-participant design with four measures of time estimation. In addition, we were able to test in a single experimental protocol the predictions of a foundational meta-analysis on the differential effects of cognitive load on prospective and retrospective duration judgments^37^. Finally, because the data were collected during the Covid-19 pandemic, we explored how lockdowns affected participants’ experience of time on the scale of a few seconds to minutes, both prospectively and retrospectively, and under controlled cognitive load.

Our study provides fresh insights for time research by assessing whether online experiments manipulating cognitive load produce effects comparable to controlled laboratory settings. Additionally, we provide intra-individual assessments of retrospective and prospective time estimation under similar cognitive load and comparisons of duration and speed of passage of time judgments. Last, we provide a historical exploration of the effects of lockdown on classical measures of time estimation that complements existing observations e.g.^2,38–40^.

## Materials and methods

### Participants

All participants in the Blursday study^2^ signed a written informed consent in adherence to the Declaration of Helsinki (2018) and the Research Ethics Committee (CER) of the Université Paris-Saclay, which approved the international study (CER-2020–020, Saclay, France). Additionally, the experimental protocol was carried out in accordance with the relevant ethical guidelines and regulations of each country^2^.

Participants completed a series of questionnaires before taking part in the cognitive test battery. Those reporting drug use and psychiatric disorders were a priori excluded from the data collection; additionally, some of the included questionnaires allow for the assessment of depression, stress, anxiety, and attenuated symptoms of psychosis. For more details, see the Blursday database article^2^. In the present study, we analyzed data from participants who completed the first n-back task during the first lockdown (lockdown group) and naive participants who were tested in 2021 outside of the lockdown (naive control group).

The lockdown group counted 1100 unique participants (361 males; mean age = 36.3 ± 15.0 y.o; demographics for 56 participants missing) from eight different countries (Argentina, Canada, France, Japan, Germany, Greece, India, Italy and Turkey) who took part in the study during the first lockdown in 2020. Prior to our main analysis, we performed data quality check and excluded 23.2% of the trials for the retrospective dataset and 19.4% for the prospective dataset. These exclusions were due to poor performance, lack of compliance to the task instructions or aberrant performance and time estimates. We provide a detailed account of criteria in the ‘Outliers’ section. Hence, we analyzed 845 retrospective trials from 845 participants (281 males; mean age = 35.0 ± 14.4 y.o.; demographics data missing from 48 participants). In the prospective tasks, we analyzed 1,063 participants (350 males; mean age = 36.1 ± 14.8 y.o.; demographics data missing from 54 participants) amounting to a total of 11,841 prospective trials.

In the control group, 199 unique participants (57 males; mean age = 32.3 ± 14.4 y.o; demographics data missing from 1 participant) from three countries (France, Japan, and Italy) participated in the study. Using a similar approach as for the lockdown group, 25% of the retrospective trials and 19.5% prospective trials were discarded (see ‘Outliers’ section). Hence, we analyzed 149 retrospective trials from 149 participants (44 males; mean age = 31.7 ± 13.3 y.o.; demographics data missing from 1 participant). In the prospective task, we analyzed 192 participants (56 males; mean age = 32.1 ± 14.2 y.o.; demographics data missing from 1 participant) amounting to a total of 2,842 prospective trials.

### Experimental design/procedure

The experiment was coded using the online research platform Gorilla™. The code for running the tasks is open source and accessible in multiple languages^2^. Our study involved three experimental tasks tested in succession: an n-back working memory task, a verbal duration estimation task, and a speed of the passage of time judgment task (PoT).

Participants were initially presented with the instructions explaining the n-back WM task, followed by a training sequence that provided performance feedback. The n-back task (Fig. 1a) presented participants with a sequence of visual letters on a white background. One n-back trial was thus a sequence of letters. In an n-back task, participants must respond whether the letter on display is identical or different from the previous one (1-back) or from the third letter before this one (3-back). The case of the letter did not matter so that a “T” or a “t” designated the same letter for the purposes of the n-back task. The letter streams were built prior using a random number generator and placing target letters in a pseudo-random position to ensure uniform allocation of attention throughout the task.

This task was built upon the original study by Polti et al. (2018) in which participants responded only when the letter was the “same” (hence, they only responded to the detected target). Herein, participants were instructed to provide a response for each letter displayed on the screen. “Same” responses were registered with the left arrow and “different” responses, with the down arrow.

The n-back trials could last 45 s or 90 s. The inter-letter intervals could be 1500 ms or 1800 ms. The 45 s sequence included 25 letters for the 1800 ms ILI or 30 letters for the 1500 ms ILI (including 6% to 24% of targets). The 90 s sequence included 50 letters or 60 letters (including 8% to 30% of targets). Each letter was presented for 500 ms. Hence, participants had 2000 ms (when the ILI was 1500 ms) or 2300 ms (when the ILI was 1800 ms) to respond to each letter.

The full within-participant experimental design was a 2 (n-back: 1 or 3) by 2 (sequence duration: 45 s or 90 s) by 2 (ILI: 1500 ms or 1800 ms) design. A summary table is provided in Fig. 1b. The between-participant design was ruled by a latin-square randomization. Additionally, this experimental design allowed calculating the full range of possible responses participants could give (Hits, Correct Rejections, Misses and False Alarms) and applying principles of signal detection theory (Green & Swets, 1966) to estimate the d prime (*d'*) and the bias ($\beta$) as psychophysical measures (Fig. 1c).

After completing a single n-back sequence, participants were prompted to estimate its duration in the format of “minutes:seconds”. According to the early internal clock proposal by Treisman (1963), verbal labels are retrieved from long-term memory storage. While verbal estimates necessitate a symbolic coding of episodic time, verbal estimation and time reproduction generate similar outcomes^41,42^. Following participants’ verbal estimation of duration, they were asked to indicate using a Likert scale whether they perceived the time during the n-back task as “Very Slow”, “Slow”, “Neutral”, “Fast”, or “Very Fast”.

The duration and speed of the passage of time judgments collected after the very first n-back trial constituted our retrospective task: participants were not aware that they would be asked to estimate duration and the speed of the passage of time. Participants were instructed to perform a single-task (n-back) with no explicit orientation to time. In the subsequent n-back trials, the duration and the speed of the experienced passage of time judgments were prospective: participants knew they would be prompted with time estimations following each n-back sequence, creating a dual-task experiment.

### Statistical analyses

All statistical analyses were carried out in the R programming language^43^ and RStudio environment^44^, using the MASS^45^, lme4^46^ and emmeans^47^ software packages.

### Signal detection analysis in the n-back task

We used Signal Detection Theory to assess participants’ performance in the n-back task using the number of *hits*, *correct rejections*, *false alarms* and *misses*. The Hit Rate (HR) was defined as the proportion of target letters participants accurately detected as target (by pressing the left arrow). The False Alarm rate (FA) was defined as the proportion of non-target letters participants incorrectly detected as target (by pressing the left arrow). HR and FA were calculated from participants’ answers on each n-back sequence.

$d{\prime}$ was calculated to ensure that participants performed the n-back task properly and it informed on participants’ ability to maximize HR and to minimize FA^36,48^: $d^{\prime } = Z\left( {HR} \right){-}Z\left( {FA} \right)$ with Z as the z-score, passing the observed proportions (HR and FA) through the normal cumulative distribution function to determine what the underlying quantile of each proportion was. Here,$d{\prime}$ corresponds to the ability to discriminate between target and non-target letters during n-back. $d{\prime}$ has previously been used to define outliers and assess performance in WM tasks^36^.

Additionally, we computed the response bias, which measures the tendency to give one response over another. The bias was computed as $\log \left( \beta \right) = \frac{1}{2}\left( {z\left( {HR} \right) - z\left( {FA} \right)} \right)$. A positive bias is a conservative tendency in which participants tend to categorize a letter as a non-target, whereas a negative bias is liberal and corresponds to the tendency to categorize letters as target.

For the retrospective tasks, each individual was thus characterized by a unique $d{\prime}$ and *bias*, accounting for their sensitivity and response bias to the very first n-back sequence they encountered (whether it was a 1-back or a 3-back), respectively. For the prospective tasks, each individual was characterized by one $d{\prime}$ and *bias* for each n-back sequence following the first one.

Participants’ response times (RT) in each experimental condition were also quantified as the mean response times to each letter in the sequence (both targets and non-targets). Accuracy, rather than speed, was emphasized in the instructions.

### Retrospective and prospective duration estimation

The retrospective duration estimates were collected after the very first n-back trial; thus, we collected a single trial per participant. The prospective duration estimates were collected following all remaining n-back trials.

In both retrospective and prospective duration estimation, we computed the relative duration taken as the ratio between the verbal estimation (s) and the veridical duration of the task (s). We proceeded in this manner to allow the direct comparison of the 45 s and 90 s duration estimates. We will refer to these measures as rDE (relative duration estimates) which are unitless. A rDE value superior to 1 indicates an overestimation of the veridical duration. A rDE value inferior to 1 indicates an underestimation of the veridical duration.

When indicated, we performed a post-hoc non-parametric Wilcoxon signed-rank test to better illustrate the interaction effects.

### Retrospective and prospective speed of passage of time judgment (PoT)

Following the same approach, PoT were partitioned between the very first n-back trial (retrospective PoT) and all subsequent n-back trials (prospective PoT). PoT were measured using a Likert scale with five different ratings displayed from left to right as: “Very Slow”, “Slow”, “Normal”, “Fast”, or “Very Fast”.

### Outliers

We identified outliers based on several criteria. First, we verified that participants were effectively doing the task: trials with a d’ inferior or equal to 0 were excluded, as they indicated the individual’s inability to discriminate a target from a distractor (HR = FA), or the tendency to classify any letter as a target (FA > HR) or as a distractor (HR < FA). This first criterion led to the exclusion of ~ 21% of all data points (227 out of 1100) for the retrospective trials, and ~ 17% of all data points (2487 out of 14,691) for the prospective trials. In the control session, we excluded ~ 21% (42 out of 199) and ~ 24% (598 out of 2529) of the retrospective trials and the prospective trials, respectively.

Subsequently, we defined outliers based on timing errors in duration estimates, which we calculated as the difference between participants’ duration estimates and the veridical duration. We computed separate z-scores (the distance of the value from the mean expressed in standard deviation) for the duration estimates obtained following the 45 s and 90 s n-back trials for each participant. Timing errors beyond two standard deviations (i.e., |zscore|> 2) away from the mean of the distribution were removed. This procedure led to the removal of ~ 3% of the total data points (28 out of 873) for the retrospective and prospective trials (363 out of 12,204). In the control session, we removed 5% (8 out of 157) and ~ 3% (89 out of 2931) of the retrospective and prospective trials, respectively.

### Linear regression and linear mixed-effect regression models

In the analysis of retrospective duration estimates (rDE), we used linear models to assess the effect of the three dependent variables (n-back sequence duration (2: 45 s, 90 s), WM load (2: 1-back, 3-back) and ILI (2: 1500 ms, 1800 ms)). We incrementally added participants’ n-back performance separately quantified by *d’* and log(*β*) to assess whether each was a good predictor of rDE in the retrospective condition.

To analyze prospective duration estimates, we used linear mixed effect models. Linear mixed effect (lme) or multilevel models are an extension of simple linear models that are most frequently used to address hierarchical and non-independent structures with multiple levels. In our case, using a mixed model helped accounting for task-level and participant-level variability. Each observation was taken into account and the interindividual variability was treated as a random effect ^49^. In addition to setting one intercept per participant, we tested random slopes to account for the effects of the independent variables (n-back load, sequence duration and ILI) on individuals’ rDE in prospective condition.

The goodness-of-fits and the significance levels were assessed using the Akaike Information Criterion (AIC) and the likelihood ratio test through Chi square (χ^2^). The AIC is a measure that helps comparing models by taking into account both likelihood and degrees of freedom. This procedure ensures that the model achieves the best fit to the data with the minimum number of predictors. The lowest AIC corresponds to the best balance between a good fit of the model to the data, and its parsimony (number of free parameters). Additionally, the likelihood ratio test determines whether adding complexity to the model makes it significantly more accurate. The likelihood ratio test (LRT) compares two hierarchically nested models, with one more complex than the other considered the nested one. The best model using the likelihood measure is defined by a significant χ^2^ test (Pr (.Chisq)), comparing one model in the list to the next. Hence, these two procedures are complementary: AIC enables comparing models with similar complexity and LRT helps in deciding between two models of fairly similar AIC. The full description of each model selection is provided in Supplementary Tables 1, 2, 3, 4 and the selected models are provided in Table 1.

**Table 1:** Best fitting models following Akaike selection and Likelihood ratio t. WM load stands for the n-back value (2: 1-back or 3-back); Duration stands for WM sequence duration (2: 45 s or 90 s). ILI is inter-letter-interval (2: 1500 ms or 1800 ms). Direction (2: prospective or retrospective). Session (2: in and out of lockdown). : denotes crossed effect (interaction only). *denotes crossed effect and main effects of both factors.

Dependent variable	Factors	Random factors	N (number of trials)	df	REML (restricted maximum likelihood)
d’	WM load + ILI + Duration + Duration:Direction		15,677	13	41,665
log(β)	ILI * WM load * Duration + Direction * Session + Direction:Duration + Direction:ILI + Direction:WM load	WM Load + Direction | Participant WM load | Participant	15,677	18	47,884
RT	Duration * WM load + ILI + ILI:WM load + Session + Direction + Direction:WM load + Direction:ILI	WM Load * Duration | Participant	15,677	21	189,074
Retro	WM Load * d’ + Session		994	6	–
Pro	Duration + WM Load + d’ + Bias + Session + d’:WM Load + d’:Duration	Duration | Participant	14,682	12	14,936
Simplified	WM Load * Direction	1 | Participant	15,677	6	16,665
Global	Duration + WM Load + ILI:WM Load + d’*Bias + d’:Duration + d’:WM Load + Direction:Duration + Session	Duration + Direction | Participant	15,677	20	15,978
Retro	Duration + WM Load		987	7	–
Pro	Duration + WM Load + Bias + d’	1 | Participant	14,633	10	–
Global	Duration + ILI + WM Load + Bias + Direction + Session:Duration + Session:WM Load + Direction:Duration	1 | Participant	15,620	14	–
PoT versus rDE	rDE + rDE:Direction	1 | Participant	15,620	7	–

### Ordinal logistic regression and mixed effect ordinal logistic regression

For the PoT ratings, we used an ordinal logistic regression for the retrospective rating (1 trial per participant) and a mixed effect logistic regression for the prospective ratings. Ordinal logistic regressions are used to determine the relationship between a set of predictors and an ordered factor dependent variable, here the Likert rating scale. The Likelihood ratio test and Akaike criterion were used in the same way as for the linear regressions of rDE.

## Results

### Validation of the online working memory n-back task

First, we assessed whether our main experimental manipulations of the cognitive load using the n-back WM task was effective (Table 2). For this, we compared the measures of sensitivity *d’* and bias *log* $\left(\beta \right)$ in the 1-back and 3-back tasks across all retrospective and prospective trials and experimental sessions (in and out of lockdown combined).

**Table 2:** All experimental manipulations significantly affected participants’ sensitivity in WM task. Results of the linear mixed effect model for *d’*, Bias and RT that were chosen following AIC and LR test, including all data from the control and lockdown sessions (Control session and Lockdown session, respectively; 15,677 obs.). : denotes crossed effect (interaction only).

Predictor	Coefficient	SD	95%	t
			Lower/Upper	
Sensitivity (d’)				
Intercept WM Load Duration ILI Direction Direction:Duration	3.10 *** -1.76 *** -0.50 *** 0.12 *** -0.38 *** 0.21 ***	0.03 0.02 0.01 0.01 0.04 0.06	+ 3.04 / + 3.15 − 1.80 / − 1.71 − 0.53 / − 0.47 + 0.09 / + 0.14 − 0.46 / − 0.29 + 0.09 / + 0.32	114.72 − 72.05 − 36.14 8.80 − 8.82 3.53
Bias (log(β))				
Intercept WM Load Duration ILI Direction Session Direction:WM Load Direction:Duration Direction:ILI Duration:ILI Duration:WM Load WM Load:ILI WM Load:ILI:Duration Session:Duration	0.88 *** -0.38 *** 0.61 *** -0.30 *** -0.71 *** 0.15 ** 0.49 *** 0.20 ** -0.17 * 0.17 *** -0.42 *** 0.28 *** -0.14 * -0.14 **	0.03 0.04 0.03 0.03 0.07 0.05 0.07 0.07 0.07 0.04 0.05 0.05 0.07 0.04	+ 0.82 / + 0.94 − 0.46 / − 0.31 + 0.54 / + 0.68 − 0.37 / − 0.24 − 0.85 / − 0.58 + 0.05 / + 0.25 + 0.35 / + 0.63 + 0.05 / + 0.34 − 0.32 / − 0.03 + 0.08 / + 0.26 − 0.51 / − 0.33 + 0.19 / + 0.37 − 0.27 / − 0.01 − 0.22 / − 0.05	28.63 − 9.97 18.32 − 9.28 − 10.45 3.00 6.78 2.71 − 2.42 3.85 − 8.86 5.85 − 2.04 − 3.26
Response time (RT)				
Intercept WM Load Duration ILI Direction Session Direction:WM Load Direction:ILI Duration:WM Load WM Load:ILI	527.41*** 54.24*** -6.51*** 14.67*** 55.35*** -23.91** -33.36*** 28.64*** -17.37*** 8.48**	3.47 3.89 1.84 1.90 5.20 7.92 8.32 7.08 2.79 2.80	+ 520.61 / + 534.20 + 46.61 / + 61.87 − 10.13 / − 2.89 + 10.95 / + 18.39 + 45.13 / + 65.54 − 39.44 / − 8.36 − 49.68 / − 17.06 + 14.72 / + 42.58 − 22.84 / − 11.90 + 2.99 / + 13.97	152.16 13.94 -3.53 7.73 10.65 -3.02 -4.01 4.04 -6.22 3.03

***p < 0.001. **p < 0.01. *p < 0.05.

**Figure 2:**
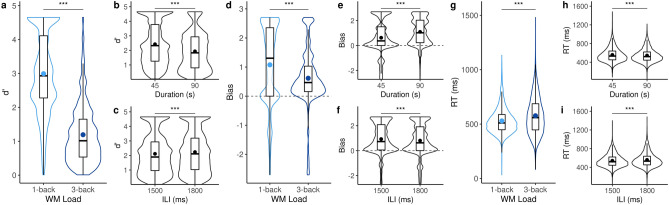
Performance in the n-back WM task. (**a**–**c**) Violin plots report the sensitivity (*d’*) in WM task as a function of WM load (**a**), Duration (**b**) and ILI (**c**). All three factors significantly impacted sensitivity. (**d**–**g**) Violin plots depict the bias (log(*β*)) as a function of WM load (**d**), Duration (**e**) and ILI (**f**). All three factors significantly impacted the bias. (**g**–**i**) Violon plots report response times (RTs) as a function of WM load (**g**), Duration (**h**) and ILI (**i**). All three factors significantly affected RTs. Statistical model provided in Table 2. ***p < 0.001.

As predicted, we found a main effect of WM memory load manipulation (1 vs. 3) on *d’*: participants’ ability to discriminate in the 1-back (*d’* = 2.77 ± 0.03) was significantly higher than in the 3-back task (*d’* = 1.01 ± 0.02; *coeff* =  − 1.76, 95% CI = [− 1.80, − 1.71]; Fig. 2a). This indicates that the main cognitive load manipulation was effective in the online experiment.

Additionally, we found a main effect of the duration of the n-back trial on *d’* (*coeff* =  − 0.50, CI = [− 0.54, − 0.47]; Fig. 2b) so that participants’ sensitivity was higher in the 45 s than in the 90 s task. Furthermore, the ILI significantly impacted the *d’* (*coeff* = 0.12, CI = [0.09, 0.14]): the shorter the ILI (i.e., the faster the rate of letter presentation), the smaller participants’ sensitivity (Fig. 2c).

Participants’ bias was significantly impacted by all experimental manipulations (Fig. 2d-f). Participants were overall conservative in their responses—i.e., biased towards reporting that a letter was not a target—with a significant positive bias (*intercept* = 0.88, CI = [+ 0.82/ + 0.94]; Fig. 2d). We found a main effect of the sequence duration on participants’ bias (*coeff* = *0.61*, CI = [+ 0.54, + 0.68]; Fig. 2e), so that participants were more conservative in the 90 s than in the 45 s trials. Participants were also significantly more conservative in the 1-back than in the 3-back task (*coeff* =  − 0.38, 95% CI = [− 0.46, − 0.31]). Finally, faster ILI significantly increased participants’ conservative bias as compared to the 1800 ms ILI (*coeff* =  − *0.30*, CI = [− 0.37, − 0.24]; Fig. 2f).

All experimental manipulations significantly affected RTs (Table 2; Fig. 2g,h,i): participants were faster in the 1-back than in the 3-back task (Fig. 2g), and faster in the 90 s as compared to the 45 s trials (Fig. 2h). Interestingly, the ILI also affected participants’ RTs so that faster ILIs significantly fastened RTs by 14.67 ms as compared to slower ILIs (95% CI = [10.95, 18.39]), suggesting a possible behavioral entrainment to the rate of letter presentation (Fig. 2i).

Altogether, our observations indicate that n-back WM tasks administered online affected participants’ performance consistent with our expectations. Specifically, participants showed greater sensitivity and faster response times for lower WM loads compared to higher ones. Subsequently, we investigate the effect of cognitive load manipulation on duration estimation and speed of passage of time perception.

### Effects of WM load on prospective and retrospective duration estimation

In agreement with previous literature, our first working hypothesis was that WM load would affect prospective duration estimation. However, we found no main effects of WM load on relative duration estimates (rDE; Table 3, Prospective).

**Table 3:** Results of the linear models that best explain the variance of the relative Durations Estimates (rDE) in the retrospective (lm, 994 obs.) and prospective (lme, 14,683 obs.) directions, and globally by combining these two conditions (lme, 15,677 obs.). Data from the Control session and the Lockdown session were included. : denotes crossed effect (interaction only).

Predictor	Coefficient	SD	95%	t
			Lower/Upper	
Retrospective				
Intercept WM Load d’ Session d’:WM Load	0.91*** 0.06 − 0.03 0.18*** − 0.10*	0.05 0.06 0.02 0.04 0.04	+ 0.81 / + 1.00 − 0.07 / + 0.18 − 0.06 / + 0.00 + 0.09 / + 0.26 − 0.19 / − 0.02	18.24 0.89 − 1.68 4.06 − 2.37
Prospective				
Intercept Duration WM Load d’ Bias Session d’:Duration d’:WM Load	0.96 *** − 0.17 *** − 0.01 0.01 0.01** 0.14*** 0.01* − 0.03***	0.02 0.01 0.02 0.01 0.01 0.03 0.01 0.01	+ 0.92 / + 1.00 − 0.20 / − 0.15 − 0.02 / + 0.04 − 0.01 / + 0.01 + 0.00 / + 0.01 + 0.08 / + 0.20 + 0.00 / + 0.02 − 0.04 / − 0.01	44.96 − 13.88 0.88 − 1.07 2.80 4.57 2.27 − 3.95
Simplified				
Intercept WM Load Direction WM Load: Direction	0.90*** − 0.02*** − 0.01 0.02	0.01 0.01 0.01 0.02	+ 0.88 / + 0.93 − 0.03 / − 0.01 − 0.04 / + 0.02 − 0.03 / + 0.07	75.29 − 3.48 − 0.77 0.90
Global				
Intercept Duration WM Load d’ Bias Session ILI 1.8 s:1-back ILI 1.8 s:3-back Duration 45 s:Direction Duration 90 s:Direction Duration:d’ d’:Bias WM Load:d’	0.99*** − 0.17*** − 0.00 − 0.01** − 0.01 0.15*** 0.01 − 0.02* − 0.09*** 0.07*** − 0.01* 0.00 − 0.01	0.02 0.01 0.02 0.01 0.01 0.03 0.01 0.01 0.02 0.02 0.00 0.01 0.01	+ 0.94 / + 1.03 − 0.19 / − 0.15 − 0.04 / + 0.03 − 0.02 / − 0.00 − 0.03 / − 0.01 + 0.08 / + 0.21 − 0.01 / + 0.02 + 0.03 / − 0.01 − 0.13 / − 0.06 + 0.03 / + 0.10 + 0.00 / + 0.02 − 0.00 / + 0.01 − 0.02 / − 0.00	45.97 − 14.93 − 0.14 − 2.95 − 0.93 4.74 1.08 − 2.25 − 4.95 3.77 2.06 1.55 − 1.45

***p < 0.001. **p < 0.01. *p < 0.05.

Rather, we found a main effect of participants’ bias on rDE (*coeff* = 0.01, CI = [+ 0.00, + 0.01]). This observation indicates that the more conservative participants were during the WM task, the longer their estimated prospective durations were. We also found a main effect of task duration so that the longer the trials were, the more participants underestimated their duration (*coeff* =  − 0.17, CI = [− 0.20, − 0.15]). Additionally, we observed a significant interaction between WM load and *d’* (*coeff* = -0.03 , CI = [− 0.04, − 0.01]) indicating that, in prospective timing, participants with a high *d’* in the 3-back task underestimated the elapsed duration more than those with a low *d’* in the 1-back task.

We then turned to the effects of WM Load and associated behavioral performances on participants’ retrospective rDE (Table 3, Retrospective). As with the prospective duration estimates, we found no main effects of WM Load or *d’* on rDE but a significant interaction between these two factors (*coeff* =  − *0.10*, CI = [− 0.19, − 0.02]). Thus, and similar to the prospective duration effects, participants with a high sensitivity (*d’*) in the high load (3-back) task significantly underestimated the elapsed duration as compared to those with low sensitivity engaged in the 1-back task. However, and contrary to the prospective duration estimation, we found no evidence that participants’ bias in the WM task or the duration of the WM task significantly affected retrospective rDE.

**Figure 3:**
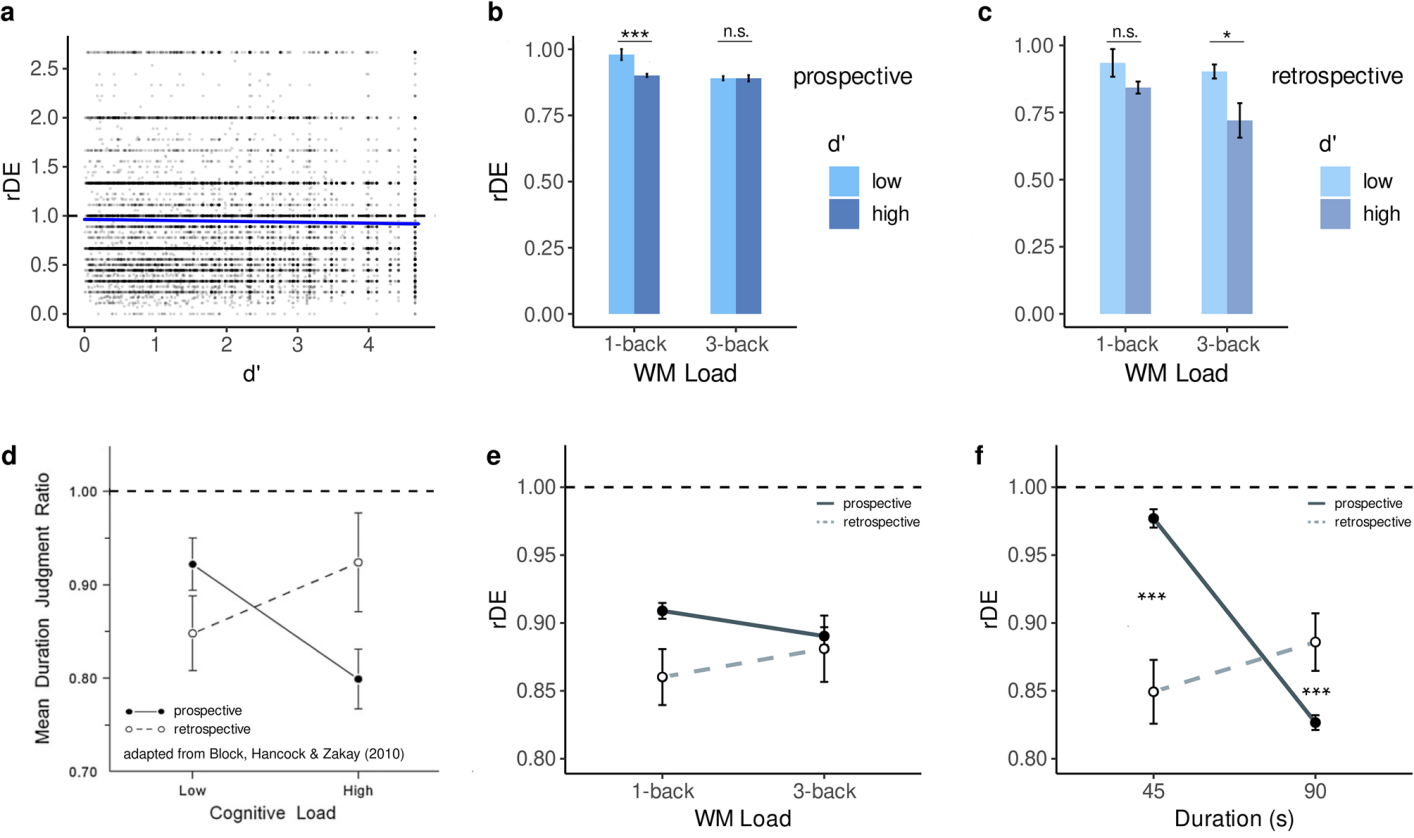
Relative duration estimates (rDE) as a function of performance in the WM task. (**a**) Relative duration estimates (rDE) in all trials (combining prospective and retrospective data) as a function of sensitivity (*d’*). Statistical model provided in Table 3. (**b**) To clarify the interaction effect in the prospective trials, we plotted the rDE in the 1-back and the 3-back WM task as a function of low and high *d’* (below or above 1.5; light and dark blue, respectively). (**c**) To illustrate the interaction effect in the retrospective trials, we plotted the rDE in the 1-back and the 3-back WM task as a function of low and high d’ (below or above 1.5; light and dark blue, respectively). In prospective duration estimation, the high WM load increases underestimation for low *d’*. In retrospective duration estimation, high WM loads affect the high *d’*. In panels (**b**) and (**c**), we report the outcomes of a post-hoc Wilcoxon test. (**d**) Like in the original meta-analysis of Block et al. (2010), (**e**) retrospective and prospective durations were underestimated (i.e. a subjective-to-objective duration ratio below one). However, and unlike the meta-analysis, the within-participant design and a replicative statistical model (see ‘﻿Methods’ section) failed to demonstrate the opposite effect of cognitive load on prospective and retrospective duration estimates. (**f**) Instead, we found that task duration showed a strong interaction with the directionality of duration estimation: prospective durations were even more underestimated with elapsing time, whereas retrospective durations tended to be less so. Statistical model provided in Table 3. *p < 0.05; ***p < 0.001; n.s.: non-significant.

Although no main effects of *d’* were found on rDE when separately considering the retrospective and prospective data, a third global model we subsequently used to contrast both time measures showed a main effect of *d’* on rDE (*coeff* = *-0.10* , CI = [− 0.02, − 0.00]; Fig. 3a) so that the higher the *d’*, the more underestimated durations tended to be.

### Prospective and Retrospective duration estimations

In their seminal meta-analysis, Block et al. (2010) showed that prospective and retrospective duration estimation yielded opposite effects as a function of the cognitive load (Fig. 3d). Herein, both prospective and retrospective tasks were used in a within-participant design, allowing the directionality (retrospective, prospective) of the rDE to be compared using a single statistical model across all trials. (Table 3, Global).

Overall, the Direction factor (2: prospective, retrospective) was not important enough to be included in our model, and no main effects of Direction could be concluded. We also found no interactions between Direction and WM load. Thus, we failed a formal replication of the meta-analysis^37^ (Fig. 3e) when considering the experimental factor WM load.

However, we found an interaction between Direction and the sequence duration. For 45 s sequence duration, retrospective rDE were lower than prospective rDE (Coeff =  − 0.09, CI = [− 0.13, − 0.06]); to the contrary, retrospective rDE were higher than prospective rDE for 90 s duration sequence (Coeff = 0.07, CI = [+ 0.03, + 0.10]; Fig. 3f).

### Speed of passage of time judgments (PoT)

#### Prospective PoT

**Figure 4:**
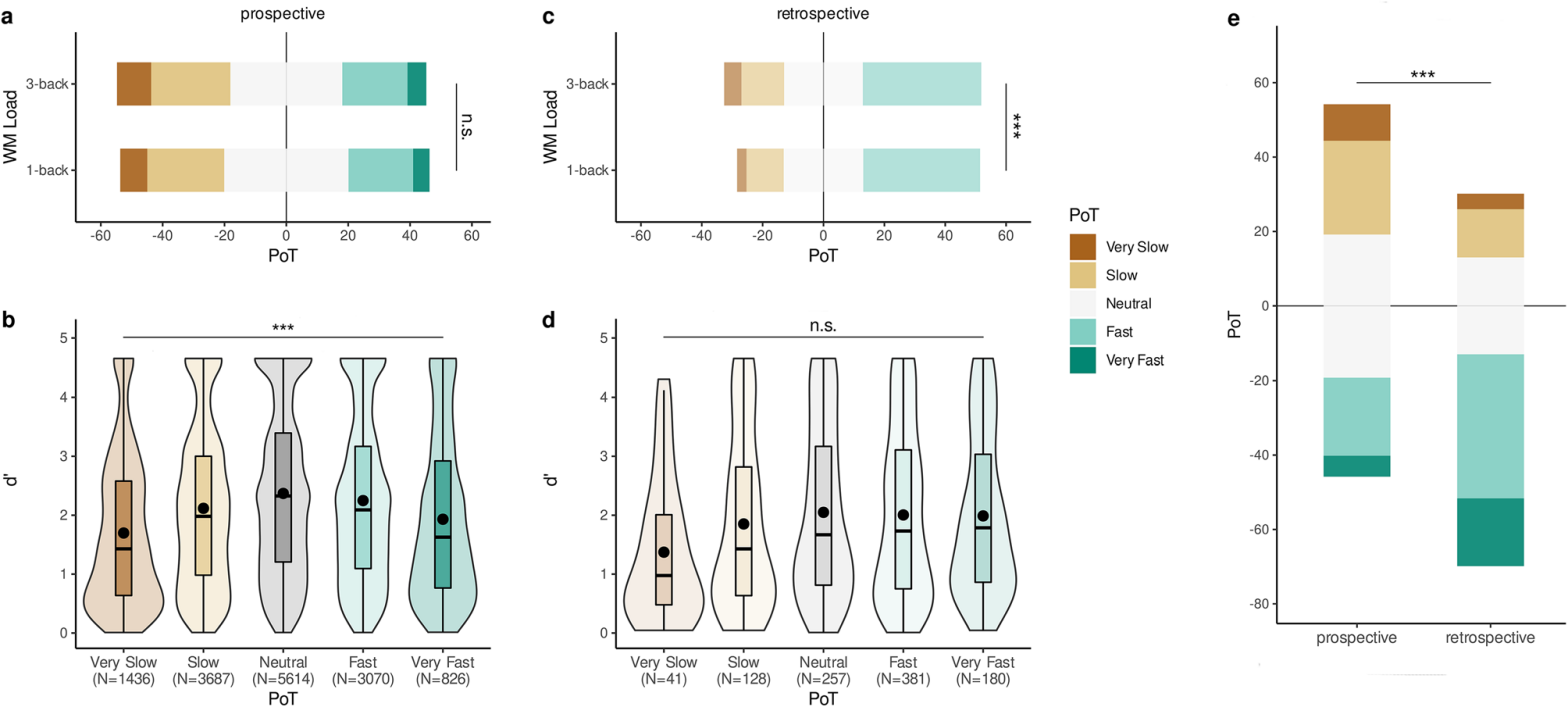
Prospective and Retrospective Speed of the Passage of Time (PoT). Prospective (**a**,**b**) and retrospective (**c**,**d**) PoT following 1-back and 3-back working memory tasks. Ratings were reported on a Likert Scale as Very Slow (brown) to Very Fast (green). (**a**) Prospective PoT were not significantly impacted by the WM Load. (**b**) Prospective PoT were significantly affected by the *d’* irrespective of the working memory load. (**c**) Retrospective PoT were significantly affected by the WM load. (**d**) Retrospective PoT were significantly affected by the *d’* irrespective of working memory load. (**e**) Overall, the speed of the experienced passage of time in prospective tasks were rated as significantly slower than in the retrospective tasks. Statistical models reported in Table 4. *p < 0.05, **p < 0.01, ***p < 0.001.

We tested whether the experimental conditions impacted participants’ prospective PoT (Table 4, Prospective). We found that the *d’,* the duration of a trial and the ILI all influenced the speed of the passage of time experienced prospectively, whereas WM load or bias did not (Fig. 4a). Specifically, we found a main effect of *d’* on prospective PoT so that for every one unit increase in *d’*, the odds to answer “Very Slow” were 11% lower, with an odds ratio of 1.11 (95% CI = [1.07, 1.15]). Thus, the more sensitive participants were in the WM task, the faster time appeared to pass (Fig. 4b).

**Table 4:** Results of the models that best explains the variance of the PoT in prospective (clmm, 14,633 obs.) and retrospective (polr, 987 obs.) direction, and globally by combining these two conditions (lme, 15,620 obs.). Data from the Control session and the Lockdown session were included. ***p < 0.001. **p < 0.01. *p < 0.05.  : denotes crossed effect (interaction only).

Predictor	Coefficient	SD	95%	t	Odds ratio	95%
			Lower/Upper			Lower/Upper
Prospective						
Duration ILI d’ ILI:d’ Session	− 1.20*** − 0.15* 0.10*** − 0.08*** 0.33*	0.03 0.06 0.02 0.02 0.15	− 1.27 / − 1.13 − 0.27 / − 0.03 + 0.07 / + 0.14 − 0.13 / − 0.04 + 0.03 / + 0.63	− 35.10 − 2.39 5.81 − 3.55 2.18	0.30 0.86 1.11 0.92 1.39	0.28 / 0.32 0.76 / 0.97 1.07 / 1.15 0.88 / 0.96 1.03 / 1.88
Retrospective						
Duration WM Load ILI	− 0.57*** − 0.26*** − 0.33**	0.12 0.12 0.12	− 0.80 / − 0.34 − 0.49 / − 0.03 − 0.56 / − 0.10	− 4.87 − 2.18 − 2.83	0.57 0.77 0.72	0.45 / 0.71 0.61 / 0.97 0.57 / 0.90
Global						
Duration ILI WM Load Bias Direction Duration45s:Session Duration90s:Session WM Load: Session Duration90s:Direction	− 1.13*** − 0.33*** − 0.17*** − 0.04** 1.40*** 0.37 * 0.09 0.17* 0.37**	0.04 0.03 0.04 0.01 0.10 0.16 0.16 0.08 0.14	− 1.21 / − 1.06 − 0.39 / − 0.27 − 0.24 / − 0.10 − 0.07 / − 0.01 + 1.21 / + 1.60 + 0.06 / + 0.68 − 0.22 / + 0.40 + 0.01 / + 0.33 + 0.10 / + 0.64	− 30.54 − 10.73 − 4.77 − 2.93 13.95 2.34 0.58 2.10 2.67	0.32 0.72 0.84 0.96 4.08 1.45 1.09 1.18 1.45	0.30 / 0.35 0.67 / 0.76 0.79 / 0.90 0.93 / 0.99 3.35 / 4.96 1.06 / 1.97 0.80 / 1.49 1.01 / 1.39 1.10 / 1.90
PoT versus rDE						
rDE rDE:Direction	− 0.72*** 1.26***	0.04 0.07	− 0.80 / − 0.63 + 1.13 / + 1.40	− 17.34 18.57	0.49 3.54	0.45 / 0.53 3.10 / 4.04

A main effect of the sequence duration was also observed so that the likelihood of answering “Very Slow” was 70% higher after the 90 s trials as compared to the 45 s trials (OR = 0.30, CI = [0.28, 0.32]). The ILI was also a good predictor of the experienced PoT: slower ILI resulted in slower PoT, so the likelihood of answering “Very Slow” after a 1.8 s ILI trial was 14% higher than after a 1.5 s ILI trial (OR = 0.86, CI = [0.76, 0.97]).

Last, we found an interaction between *d’* and ILI: the odds of answering “Very Slow” were 8% higher for every one unit increase in *d’* following 1.8s ILI trials (OR = 0.92, CI = [0.88, 0.96]).

#### Retrospective PoT

All three experimental conditions influenced the retrospective experience of the speed of the passage of time (see Table 4, Retrospective). Unlike the prospective PoT, a main effect of WM Load was found on the retrospective PoT: the likelihood of responding “Very Slow” was 23% higher for 3-back than for 1-back trials (OR = 0.77, CI = [0.61, 0.97]; Fig. 4c). Intriguingly, no main effect of *d’* or bias were found (Fig. 4d). A main effect of duration on retrospective PoT showed that the odds of answering “Very Slow” following a 90 s trial were 43% higher than following a 45 s trial (OR = 0.57, CI = [0.45, 0.71]). Participants had a 28% greater chance to judge that time passed “Very Slow” for trials with the slowest 1.8 s ILI (OR = 0.72, CI = [0.57, 0.90]).

#### Prospective and retrospective PoT

We compared the retrospective and prospective experience of the speed of passage of time using a global model that incorporated all trials (Table 4, Global) for both sessions (Lockdown and Control) and directions (prospective, retrospective). We found a main effect of Direction on PoT: participants rated time as passing much faster during retrospective compared to the prospective trials (Fig. 4e): the likelihood of answering “Very Slow” was approximately four times higher for prospective trials than for retrospective trials (OR = 4.08, CI = [3.35, 4.96]). We also found an interaction between trial Duration (2: 45 s, 90 s) and Direction so that the odds of answering “Very Slow” in the 90 s duration was 45% higher for prospective than for retrospective trials (OR = 1.45, CI = [1.10, 1.90]).

#### Relation between duration estimation and the experienced speed of passage of time

**Figure 5:**
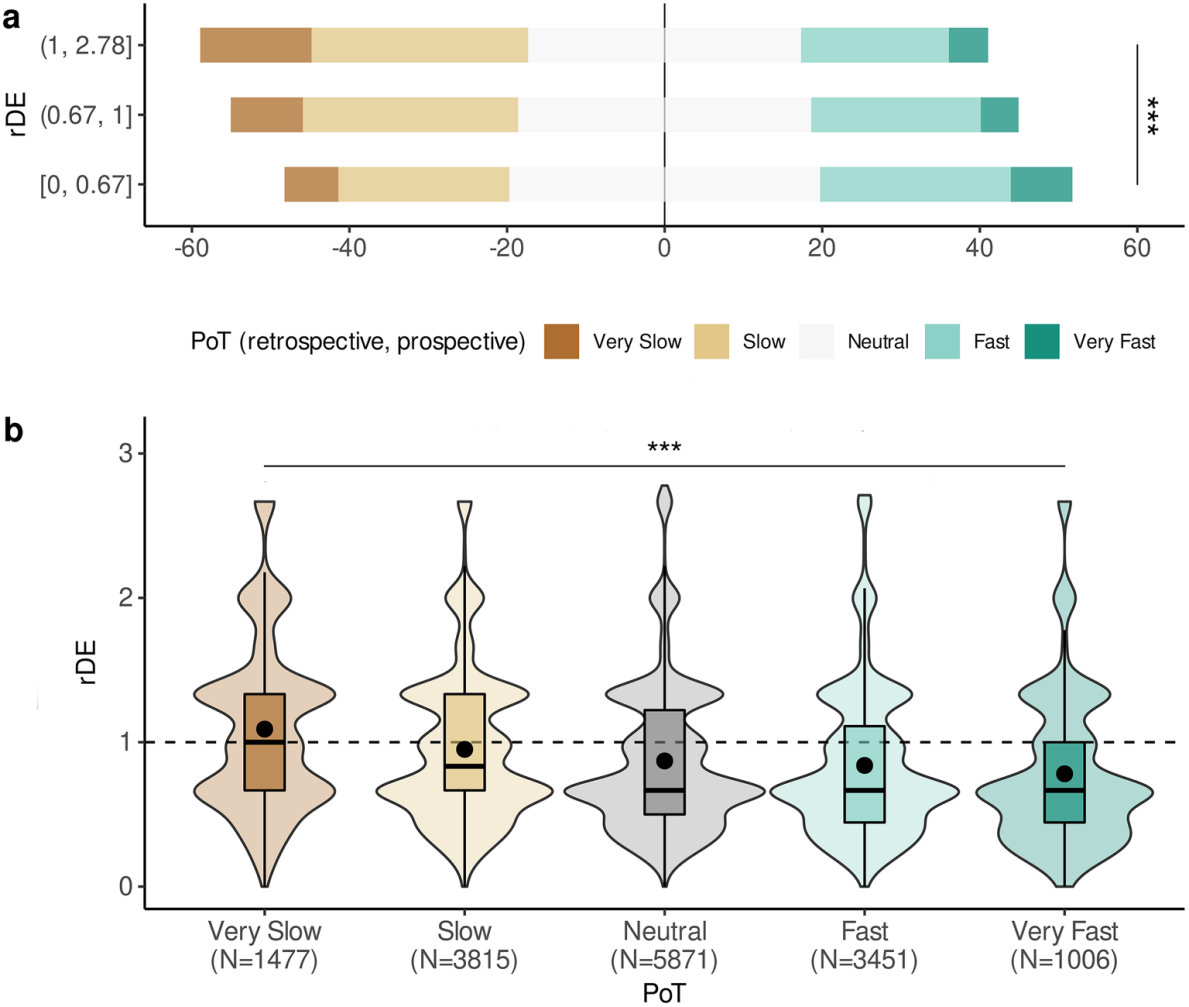
Relation between duration estimation and passage of time ratings. Each n-back trial was associated with a duration estimate (rDE) and a PoT. Retrospective and prospective durations were pulled together. (**a**) Proportion of PoT ratings for each rDE tertile. By setting the 0 of the x-axis on the “Neutral” answer in PoT, diverging stacked bar allow to see whether participants tended to rate the passage of time as slow or fast according to the rDE tertile (1st = [0, 0.667], 2nd = (0.667, 1], 3rd = (1, 2.78]). As predicted, the slower the PoT (dark brown, top row), the longer the rDE (3^rd^ tercile, top row), and the faster the PoT (dark green, bottom row), the shorter the rDE (1^st^ tercile, bottom row). (**b**) The distribution of each PoT as a function of rDE. rDE = 1 is the veridical estimate. Statistical model reported in Table 4. ***p < 0.001.

Last, we asked whether duration and speed estimates were related. For this, we tested the rDE quantified in the duration estimations as a predictor of the PoT judgments for all trials (Table 4). We found that participants who overestimated the duration (rDE > 1) rated the passage of time as passing “Slow” or “Very Slow” (Fig. 5): the likelihood of answering “Very Slow” was 51% higher for every unit increase of rDE (OR = 0.49, CI = [0.45, 0.53]). Additionally, we found a significant interaction between rDE and Direction: for every one unit increase in rDE, participants were ~ 3.5 times less likely to respond “Very Slow” in retrospective than in prospective trials (OR = 3.54, CI = [3.10, 4.04]).

### Effects of lockdown on information-theoretic measures of time perception

#### Lockdown affects prospective and retrospective duration estimations

**Figure 6:**
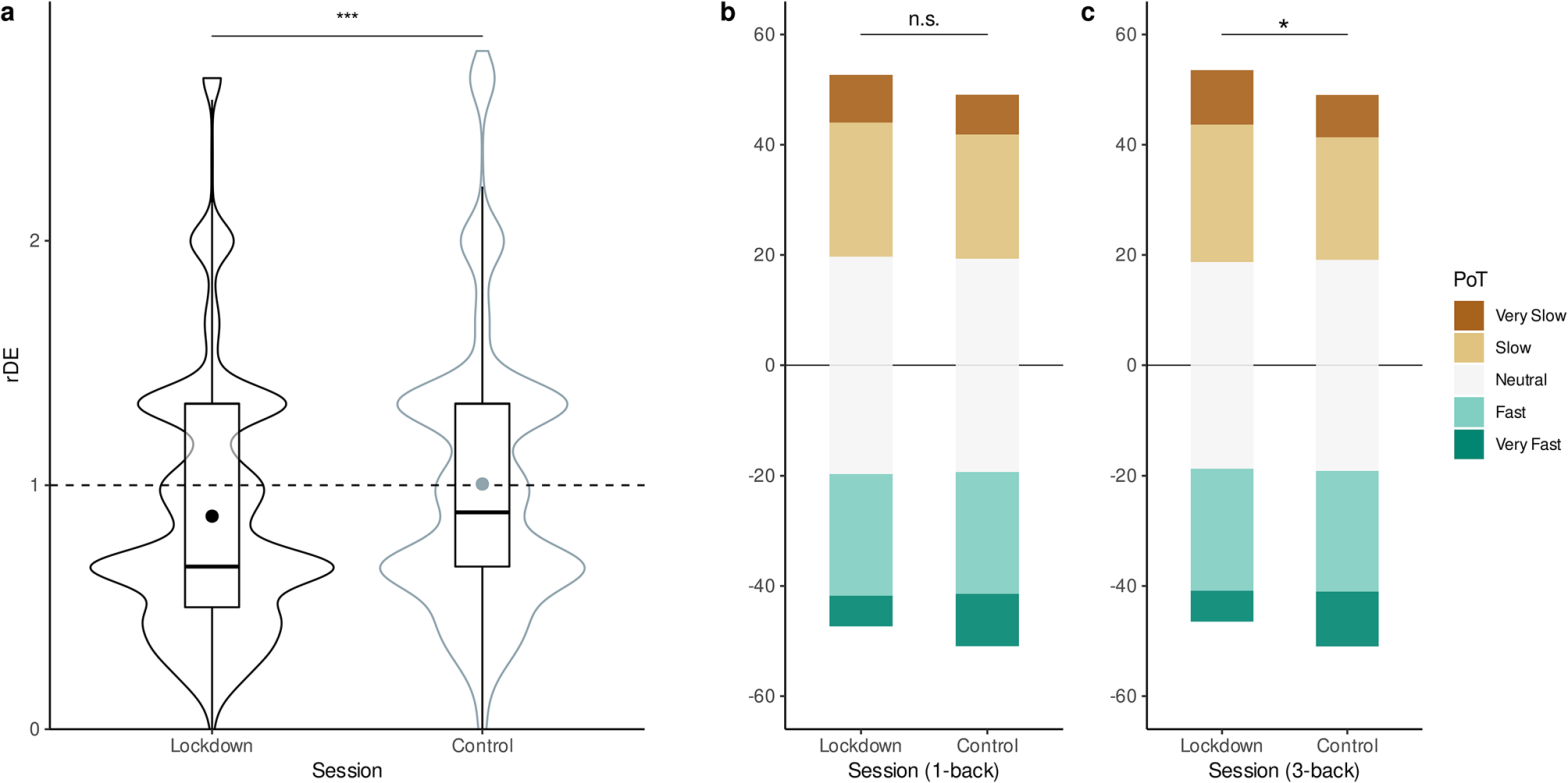
Effect of lockdown on time perception under controlled cognitive load. (**a**) Violin plots showing the distribution of the rDE during the Lockdown session and during the Control session (dark and light gray, respectively). Overall, when participants were performing a working memory task, durations tended to be underestimated during lockdown as compared to outside of it. (**b**) A diverging stacked bar shows the proportion for PoT during Lockdown (left) and in the Control session (right). Overall, participants performing a 3-back tasks estimated time to go slower during lockdown than outside of it. Statistical model provided in Table 3 and 4. *p < 0.05; ***p < 0.001.

We found a main effect of Session (2: Lockdown, Control) on rDE of 0.15 (95% CI = [+ 0.08, + 0.21]) indicating that during the Control session, participants’ estimations of duration were on average very close to the objective task duration (rDE = 0.99 ± 0.02) whereas during the Lockdown, participants significantly underestimated durations (rDE = 0.84 ± 0.02). This effect (Fig. 6a) corresponded to participants underestimating the 45 s trials by 6.75 s and the 90 s trials by 13.5 s during the lockdown as compared to outside of it. We found no other interactions with the Session factor.

#### Effects of lockdown on PoT

We directly compared participants’ PoT collected during the Lockdown and during the Control sessions (Fig. 6b,c) using the same model as above (see Table 4, Global). We found no main effects of session on PoT.

However, a significant interaction between Session and WM Load was found following 3-back sequences: the likelihood of answering “Very Slow” during the Lockdown was 18% higher than in the Control session (OR = 1.18, CI = [1.01, 1.39]; Fig. 6c). Additionally, a significant interaction between Duration and Session was found: the odds of answering “Very Slow” was 44.7% higher during the Lockdown session than during the Control session for the shortest 45 s sequences (OR = 1.45, CI = [1.06, 1.97]). Importantly, we found no interactions between Session and the rDE predictors, indicating that the relation between duration estimation and PoT ratings was not significantly affected by the lockdown.

## Discussion

In this study, we explored the effect of verbal working memory load on participants’ time perception using a duration estimation task and a speed of passage of time rating. We used a within-participant design to test different working hypotheses on four distinct measures of time experience (prospective and retrospective rDE and PoT) using the Blursday database^2^. An additional group of participants, tested outside of Covid-19 lockdowns, helped in assessing differences in time experiences in and out of lockdown under controlled cognitive load. Below, we discuss how separating individuals’ capacity to maintain and update information in working memory (sensitivity to the load) from their response strategy (bias) may improve assessment of cognitive interference on various measures of time experience. Additionally, we discuss the status of perceived duration and speed of time in the literature.

### Working memory task

In this online experiment, participants showed an overall better sensitivity (*d’*) in the 1-back compared to the 3-back task, suggesting that the main cognitive load manipulation (n-back) was effective: the executive cost of a lower load was lower than that of a higher load^36^. Additionally, participants exhibited a significant change in their response strategy, being more conservative in the 1-back task than in the 3-back task (Fig. 2).

Participants performed better on the shortest WM tasks (45 s) than on the longest ones (90 s). This observation could be due to uncontrolled distractions and lapses of attention during online experimentation, which may be more impactful with the lengthening of the task. We also found that the d’ was better in prospective trials than in the retrospective trials: by design, the retrospective trials were systematically the first n-back trial participants encountered in the WM task. Therefore, this effect can be attributed to familiarity and training.

Consistent with prior literature^50^, participants exhibited faster response times in the 1-back sequences compared to the 3-back sequences, despite the fact that speed was not emphasized in our instructions. Interestingly, a slower rate of letter presentation led to slower response times. This could potentially indicate an automatic sensorimotor synchronization (or entrainment) to the sequential letter presentation, and the requirement for participants to respond following each letter. Interestingly, the rate of letter presentation affected both sensitivity and response bias. Further investigation into the precise cognitive process affected by sensorimotor expectation and synchronization in this verbal WM task would be worth exploring.

The experimental design build on an existing n-back task^28^ with the introduced novelty that we required an answer for each letter. This approach enabled testing participants’ sensitivity and bias in the WM task separately^36^. Our findings suggest that participants’ sensitivity and bias may indeed provide a more comprehensive account of the impact of WM load manipulation on individuals, since they predicted several effects that were not explained by WM load only. We discuss these aspects in more details below.

### Duration estimations and task load

In two prominent models of time perception, the resource allocation model^51^ and the scalar timing model^52^, non-temporal information that is present concurrently with time estimation competes with the allocation of resources. This competing information shortens temporal estimations^37^. We successfully replicated this observation (Fig. 3).

It has been suggested that the cost associated with dual-task performance impacts duration estimation, rather than the nature of the non-timing task itself^53^. For instance, previous findings reported that neither task switching^54^, nor inhibition, or access, affected timing^31^. However, memory tasks^54^, updating^31^, and non-sequential coordinative demands^55^ have been shown to affect timing. In agreement with these observations, previous work using an n-back task differentiated the effect of attention and verbal WM load on prospective duration estimation and displayed a parametric effect of task load on the underestimation of durations^28^. Results in the literature align well with the predictions of the allocation of resources model^53^.

Unfortunately, we did not directly replicate prior work^28^ as we did not find a parametric effect of WM load on prospective or retrospective duration underestimations. One possibility is that conducting online experiments cannot provide a strict control of participants’ behavior during the task. If manipulating the n of the n-back suffices for fully controlling participants’ task load under controlled lab settings, it may be insufficient for online experimenting. Specifically, the absence of distractions in lab settings mostly insure participants’ full attention to the task whereas distractions in online experimenting may be unavoidable. Therefore, highly controlled lab settings may help stabilize inter-individual variability and experimental factors alone may serve as reliable proxies for individuals’ resource allocation. To the contrary, online experimenting may exacerbate interindividual variability due to participants not fully focusing on the task, being prone to distractions, or to the variability of the environments themselves. Therefore, in online experimenting, a more refined assessment of an individual’s resource allocation may be needed to account for experimental idiosyncrasies of uncontrolled online settings. This can be achieved using signal detection theory assessing the individual’s sensitivity and bias *in lieu of* the experimental factor alone.

In particular, the performances on the WM task enable teasing the effect of experimental factors on participants’ resource allocation. For instance, we found a systematic interaction between d’ and WM load on the estimation of prospective and retrospective durations. This suggests that considering the executive demands at the individual level may provide a better assessment of participants’ cognitive engagement in the task and of how much resources are effectively interfering with duration estimations. We found that prospective and retrospective durations were more underestimated as the *d’ *increased, particularly at the highest load (3-back). Overall, these results are consistent with the known cognitive interferences on duration estimations and the implication of updating^31^ that gives its specificity to the n-back WM task. Similarly, our study does not fully replicate the findings predicted by the meta-analysis^37^ in which the greater the cognitive load, the greater the prospective underestimation but the smaller the retrospective underestimation (Fig. 3d). We did not reproduce the reverse effect of cognitive load on prospective and retrospective timing although a trend was apparent (Fig. 3e). However, as discussed above, it is possible that the cognitive cost is not solely attributable to the WM load manipulated with the n-back task but instead, to the individuals’ resource allocation particularly in the retrospective durations (Fig. 3c).

We found an interaction between the Direction (prospective, retrospective) and the duration of n-back sequences: as the n-back sequences increased in duration, prospective estimates were lower but retrospective estimates were higher (Fig. 3f). The results for prospective timing are in line with previous observations in which underestimation appears to scale with prospective duration for a given task load^28^. Interestingly, the effect was not observed for retrospective durations. As such, the scaling may not be accounted for solely by memory or timing effects and may instead require the co-existence of a competing task load with intended timing. The effect does capture the well-known scalar properties of timing in which timing errors scale with duration^56^.

### Are duration estimations and passage of time ratings telling us the same thing?

Several recent findings suggest that passage of time judgments capture a different phenomenon than the one measured by the estimation of durations^23–25,27^. In the current analysis, we found that PoT and rDE were well correlated in that both judgments went in the expected direction: participants who overestimated the duration of an n-back trial tended to respond “Very Slow”, while those who underestimated the duration tended to report “Very Fast”.

Nevertheless, our experimental factors affected duration and speed of time ratings differently. For instance, whereas duration estimates were strongly modulated by the interaction between WM load and d’, irrespective of the direction of the judgment (retrospective or prospective), a double dissociation of task load and sensitivity (*d’*) was found in the direction of speed ratings. Specifically, retrospective speed ratings were significantly affected by WM load but not by the *d’*, while prospective speed ratings were significantly affected by the *d’* but not by the WM load. These observations suggest that participants’ task load and executive engagement systematically interfered with duration estimation, regardless of whether participants knew they had to estimate a duration or not. In other words, task demands impacted both the experience and the retrieval of duration, regardless of whether participants attended to time.

To the contrary, the direction (prospective *vs.* retrospective), and associated cognitive strategy (dual-task vs. single WM task) influenced participants’ ratings of the speed of the passage of time. Executive engagement (*d’*) had the greatest impact on prospective speed ratings, while actual WM load had the greatest impact on retrospective ratings. Thus, one possibility is that retrospective PoT were inferred from participants’ knowledge of task requirements, rather than their felt engagement in it; a different process may be involved when prospectively paying attention to time. Indeed, our observations converge with a proposal that the passage of time is a metacognitive experience^21^, which is not a felt sensory experience but rather the outcome of a metacognitive inference process relying on multiple sensory and cognitive factors. Consistent with this hypothesis, subsequent findings suggested the implication of mnemonic and attentional processes^57,58^ converging towards the notion that the experienced passage of time may capture a metacognitive aspect of temporal experience^27^. It is noteworthy that metacognitive assessments of duration and time experiences have been demonstrated^59–61^.

For both retrospective and prospective estimations, the duration of the sequence influenced the speed of the passage of time ratings, suggesting a scaling effect. Participants reported time to pass more quickly during 45 s compared to 90 s trials. This is noteworthy, as in the retrospective PoT, participants encountered the n-back task for the first time, and had no prior expectation of the duration or of their experienced passage of time during the exercise. In other words, these results are intriguing given the absence of an explicit scale or duration that participants could have used to estimate their experienced speed of the passage of time^23^. For prospective speed, participants could make comparisons between the 45 s and the 90 s trials, as they had already performed the tasks. In this situation, PoT may rely on temporal expectations and predictions^20,21,21,22,62^.

As to the relation between duration and speed of time, the retrospective trials showed a weaker relation between rDE and PoT. Participants were approximately 3.5 times less likely to respond “Very Slow” for each unit increase in rDE as compared to the prospective trials. This observation supports the notion that in real-life settings, and in the absence of orientation to time, the estimation of duration and speed of the passage of time may rely on different, but not fully independent, processes.

### Lockdown effects

Previous reports using the same sample of participants reported that duration estimations in the minutes-to-hours scales were overall closer to veridical timing during lockdown than outside of it^2^. Our study focused on shorter time scales (minute-scale) and showed that duration estimates, whether prospective or retrospective, were significantly shorter during lockdown. These observations were found under controlled task demands. On the contrary, the perceived speed of the passage of time was affected during the lockdown as participants undertaking the high load task reported time to be slower than outside of the lockdown, consistent with many recent reports^38–40^. The different trend between duration estimation and passage of time during lockdown is intriguing, but it does not override the main relation between duration and the speed of the passage of time. We also found very few interactions of lockdown with the main experimental factors (task load, ILI, duration), suggesting that none of our manipulations could easily account for the underestimation of duration during the lockdown as compared to outside of it.

## Conclusions

Our online study explored several quantitative measures of psychological time estimations while participants performed a verbal working memory task. Our results provide important observations on the relation between prospective and retrospective durations and passage of time (experienced and remembered time, respectively). The intricate relation between time estimation and the allocation of cognitive resources (attention and memory) remains open. Duration and speed of the passage of time consistently maintained the expected relationship of shorter durations feeling to pass faster. However, both psychological measures also show distinct influences from experimental manipulations. Lastly, lockdown effects overall converged with previous reports in demonstrating that the speed of time, at short time scale and when being cognitively engaged, still felt as passing slower during lockdown than outside of it.

### Supplementary Information

Supplementary Information.

## Data Availability

The full data are already fully available through the Blursday database here https://timesocialdistancing.shinyapps.io/Blursday/.
