# Supplementary material for: Cognitive effects on experienced duration and speed of time, prospectively, retrospectively, in and out of lockdown
Source: Sci Rep. 2024 Jan 23;14:2006. doi: 10.1038/s41598-023-50752-7 (PMC10805715; doi:10.1038/s41598-023-50752-7)
Supplement: Supplementary file 1 — Supplementary Information. [file 41598_2023_50752_MOESM1_ESM.pdf]

Supplementary Materials

Supplementary Table 1: Models of N-back Performance (d', Bias and RT). Choice of the best-fitting model following akaike selection and lr test.

Global d'	Df	AIC	*LogLik*	χ^2	Pr(>χ^2)
d'Global1: *d' ~ WM Load + (1 | Participant)*	4	44069.58	-22031	-	-
d'Global2: *d' ~ WM Load + ILI + (1 | Participant)*	5	44020.01	-22005	51.57	6.916e-13 ***
d'Global3: *d' ~ WM Load + ILI + Duration + (1 | Participant)*	6	42888.12	-21438	1133.9	< 2.2e-16 ***
d'Global4: *d' ~ WM Load + ILI + Duration + Direction + (1 | Participant)*	7	42818.72	-21402	71.39	< 2.2e-16 ***
d'Global5: *d' ~ WM Load + ILI + Duration * Direction + (1 | Participant)*	8	42809.56	-21397	11.16	0.00084 ***
d'Global6: *d' ~ WM Load + ILI + Duration * Direction + (WM Load | Participant)*	10	41677.98	-20829	1135.6	< 2.2e-16 ***
d'Global7: *d' ~ WM Load + ILI + Duration * Direction + (WM Load + Direction | Participant)*	13	41657.03	-20816	25.95	6.025e-06 ***
**Global Bias**	**Df**	**AIC**	***LogLik***	χ^2	**Pr(>χ^2)**
*BiasGlobal*1: *Bias ~ Duration + (1 | Participant)*	4	49405.34	-24699	-	-
*BiasGlobal*2: *Bias ~ Duration + ILI + (1 | Participant)*	5	49356.94	-24674	50.40	1.256e-12 ***
*BiasGlobal*3: *Bias~ Duration + ILI + WM Load + (1 | Participant)*	6	48731.86	-24360	627.08	< 2.2e-16 ***
*BiasGlobal*4: *Bias ~ Duration * WM Load + ILI + (1 Participant)*	7	48545.96	-24266	187.9	< 2.2e-16 ***
*BiasGlobal*5: *Bias ~ Duration * WM Load * ILI + (1 |Participant)*	10	48507.21	-24244	44.74	1.049e-09 ***
*BiasGlobal*6: *Bias ~ Duration * WM Load * ILI + Session + d':WM Load + (1 | Participant)*	11	48505.42	-24242	3.79	0.05156 .
*BiasGlobal*7: *Bias ~ Duration * WM Load * ILI + Session + Direction + (1 | Participant)*	12	48315.70	-24146	191.73	< 2.2e-16 ***
*BiasGlobal*8: *Bias ~ Duration * WM Load * ILI + Session + Direction + Session:Duration + Direction:Duration + Direction:ILI + Direction:WM Load + (1 | Participant)*	16	48261.49	-24115	62.20	9.984e-13 ***
*BiasGlobal*9: *Bias ~ Duration * WM Load * ILI + Session + Direction + Session:Duration + Direction:Duration + Direction:ILI + Direction:WM Load + (WM Load| Participant)*	18	47853.05	-23908	412.44	< 2.2e-16 ***
**Global mRT**	**Df**	**AIC**	***LogLik***	χ^2	**Pr(>χ^2)**
mRTGlobal1: *mRT ~ Duration + (1 | Participant)*	4	193093.4	-96543	-	-

mRTGlobal2: *mRT ~ Duration + ILI + (1 | Participant)*	5	192951.8	-96471	143.59	< 2.2e-16 ***
mRTGlobal3: *mRT ~ Duration + ILI + WM Load + (1 | Participant)*	6	192155.6	-96072	798.19	< 2.2e-16 ***
mRTGlobal4: *mRT ~ Duration + ILI + WM Load + Duration:WM Load + ILI:WM Load + (1 | Participant)*	8	192121.0	-96053	38.62	4.108e-09 ***
mRTGlobal5: *mRT ~ Duration + ILI + WM Load + Duration:WM Load + ILI:WM Load + Session + (1 | Participant)*	9	192112.0	-96047	10.976	0.00092 ***
mRTGlobal6: *mRT ~ Duration + ILI + WM Load + Duration:WM Load + ILI:WM Load + Session + Direction + (WM Load | Participant)*	10	191848.9	-95914	265.15	< 2.2e-16 ***
mRTGlobal7: *mRT ~ Duration + ILI + WM Load + Duration:WM Load + ILI:WM Load + Session + Direction + Direction:WM Load + (1 | Participant)*	11	191832.2	-95905	18.731	1.505e-05 ***
mRTGlobal8: *mRT ~ Duration + ILI + WM Load + Duration:WM Load + ILI:WM Load + Session + Direction + Direction:WM Load + Direction:ILI + (1 Participant)*	12	191812.8	-95894	21.349	3.827e-06 ***
mRTGlobal9: *mRT ~ Duration + ILI + WM Load + Duration:WM Load + ILI:WM Load + Session + Direction + Direction:WM Load + Direction:ILI + (WM Load | Participant)*	14	189387.9	-94680	2429	< 2.2e-16 ***
mRTGlobal10: *mRT ~ Duration + ILI + WM Load + Duration:WM Load + ILI:WM Load + Session + Direction + Direction:WM Load + Direction:ILI + (WM Load * Direction | Participant)*	21	189159.5	-94559	242.4	< 2.2e-16 ***

Supplementary Table 2: Models of relative Duration Estimation (rDE). Choice of the best-fitting model following akaike selection and lr test.

**Retrospective rDE**	**Df**	**AIC**	***LogLik***	**χ^2**	**$Pr(>\chi^2)$**
rDEretro1: *rDE ~ d'*	3	1430.3	-712.14	-	-
rDEretro2: *rDE ~ WM Load * d'*	5	1428.6	-709.28	5.72	0.05728 .
rDEretro3: *rDE ~ WM Load * d' + Session*	6	1414.1	-701.06	16.43	5.051e-05***

**Prospective rDE**	**Df**	**AIC**	***LogLik***	**χ^2**	**$Pr(>\chi^2)$**
rDEpro1: *rDE ~ Duration + (1 | Participant)*	4	15152.2	-7572.1	-	-
rDEpro2: *rDE ~ Duration + WM Load + (1 | Participant)*	5	15141.5	-7565.7	12.77	0.0003***
rDEpro3: *rDE ~ Duration + WM Load + d' + (1 | Participant)*	6	15129.6	-7558.8	13.90	0.0002***
rDEpro4: *rDE ~ Duration + WM Load + d' + Bias + (1 Participant)*	7	15125.8	-7555.9	5.75	0.0165*
rDEpro5: *rDE ~ Duration + WM Load + d' + Bias + Session + (1 |Participant)*	8	15107.9	-7546.0	19.91	8.124e-06***
rDEpro6: *rDE ~ Duration + WM Load + d' + Bias + Session + d':WM Load + (1 | Participant)*	9	15095.9	-7539.0	14.00	0.0002***
rDEpro7: *rDE ~ Duration + WM Load + d' + Bias + Session + d':WM Load + d':Duration + (1 | Participant)*	10	15091.1	-7535.5	6.85	0.0088**
rDEpro8: *rDE ~ Duration + WM Load + d' + Bias + Session + d':WM Load + d':Duration + (Duration | Participant)*	12	14895.5	-7435.8	199.53	< 2.2e-16 ***

**Global rDE**	**Df**	**AIC**	***LogLik***	**χ^2**	**$Pr(>\chi^2)$**
rDEglobal1: *rDE ~ Duration + (1 | Participant)*	4	16249.00	-8120.5	-	-
rDEglobal2: *rDE ~ Duration + WM Load + (1 | Participant)*	5	16238.71	-8114.4	12.2875	0.000456 ***
rDEglobal3: *rDE ~ Duration + WM Load + ILI:WM Load + (1 | Participant)*	7	16235.13	-8110.6	7.58	0.022546 *
rDEglobal4: *rDE ~ Duration + WM Load + ILI:WM Load + d' + (1 | Participant)*	8	16220.31	-8102.2	16.81	4.120e-05 ***
rDEglobal5: *rDE ~ Duration + WM Load + ILI:WM Load + d' + Bias + (1 | Participant)*	9	16216.55	-8099.3	5.76	0.016367 *
rDEglobal6: *rDE ~ Duration + WM Load + ILI:WM Load + d' + Bias + Session + (1 | Participant)*	10	16197.19	-8088.6	21.36	3.804e-06 ***
rDEglobal7: *rDE ~ Duration + WM Load + ILI:WM Load + d' + Bias + Session + Direction:Duration + (1 | Participant)*	12	16159.95	-8068.0	41.24	1.108e-09 ***

rDEglobal8: *rDE ~ Duration + WM Load + ILI:WM Load + d' + Bias + Session + Direction:Duration + d':Bias + (1 | Participant)*	13	16153.47	-8063.7	8.48	0.003590 **
rDEglobal9: *rDE ~ Duration + WM Load + ILI:WM Load + d' + Bias + Session + Direction:Duration + d':Bias + d':Duration + (1 | Participant)*	14	16150.80	-8061.4	4.66	0.030857 *
rDEglobal10: *rDE ~ Duration + WM Load + ILI:WM Load + d' + Bias + Session + Direction:Duration + d':Bias + d':Duration + d':WM Load + (1 | Participant)*	15	16143.53	-8056.8	9.28	0.002318 **
rDEglobal11: *rDE ~ Duration + WM Load + ILI:WM Load + d' + Bias + Session + Direction:Duration + d':Bias + d':Duration + d':WM Load + (WM Load | Participant)*	17	15952.46	-7959.2	195.06	< 2.2e-16 ***
rDEpro12: *rDE ~ Duration + WM Load + ILI:WM Load + d' + Bias + Session + Direction:Duration + d':Bias + d':Duration + d':WM Load + (Direction + WM Load | Participant)*	20	15916.59	-7938.3	41.88	4.257e-09 ***

Supplementary Table 3: Models of Passing of Time Judgment (PoTJ). Choice of the best-fitting model following akaike selection and lr test.

**Retrospective PoTJ Model (polr)**	**Df**	**AIC**	***LogLik***	**χ^2**	**Pr(>χ^2)**
PoTJretro1: PoTJ ~ *Duration*	5	2797.99	-1394.0	-	-
PoTJretro2: PoTJ ~ *Duration + ILI*	6	2791.77	-1389.9	8.22	0.004142 **
PoTJretro3: PoTJ ~ *Duration + ILI + WM Load*	7	2789.01	-1387.5	4.7532	0.02924 *
**Prospective PoTJ Model (clmm)**	**Df**	**AIC**	***LogLik***	**χ^2**	**Pr(>χ^2)**
PoTJpro1: PoTJ ~ *Duration + (1 | Participant)*	6	35383.91	-17686	-	-
PoTJpro2: PoTJ ~ *Duration + WM Load + (1 | Participant)*	7	35374.41	-17680	11.50	0.0007 ***
PoTJpro3: PoTJ ~ *Duration + WM Load + ILI + (1 | Participant)*	8	35273.38	-17629	103.03	< 2.2e-16 ***
PoTJpro4: PoTJ ~ *Duration + d' + ILI + (1 Participant)*	8	35264.06	-17624	9.32	< 2.2e-16 ***
PoTJpro5: PoTJ ~ *Duration + d' + ILI + Session + (1 |Participant)*	9	35261.33	-17622	4.73	0.02956 *
PoTJpro6: PoTJ ~ *Duration + d' + ILI +Session + d':ILI + (1 | Participant)*	10	35250.72	-17615	12.61	0.0004 ***
**Global PoTJ (clmm)**	**Df**	**AIC**	***LogLik***	**χ^2**	**Pr(>χ^2)**
PoTJglobal1: PoTJ ~ *Duration + (1 | Participant)*	6	38519.20	-19254	-	-
PoTJglobal2: PoTJ ~ *Duration + WM Load + (1 | Participant)*	7	38504.66	-19245	16.54	4.761e-05 ***
PoTJglobal3: PoTJ ~ *Duration + WM Load + ILI + (1 | Participant)*	8	38397.49	-19191	109.17	< 2.2e-16 ***
PoTJglobal4: PoTJ ~ *Duration + WM Load + ILI + Bias + (1 | Participant)*	9	38373.68	-19178	25.81	3.77e-07 ***
PoTJglobal5: PoTJ ~ *Duration + WM Load + ILI + Bias + Direction + (1 | Participant)*	10	37835.96	-18908	539.72	< 2.2e-16 ***
PoTJglobal6: PoTJ ~ *Duration + WM Load + ILI + Bias + Direction + Session:Duration + (1 | Participant)*	12	37822.71	-18899	17.251	0.0002 ***
PoTJglobal7: PoTJ ~ *Duration + WM Load + ILI + Bias + Direction + Session:Duration + Session:WM Load + (1 Participant)*	13	37820.32	-18897	4.38	0.03631 *
PoTJglobal8: PoTJ ~ *Duration + WM Load + ILI + Bias + Direction + Session:Duration + Session:WM Load + Direction:Duration + (1 |Participant)*	14	37815.19	-18894	7.13	0.007571 **
**Global PoTJ x rDE (clmm)**	**Df**	**AIC**	***LogLik***	**χ^2**	**Pr(>χ^2)**

PoTJrDE1: PoTJ ~ *rDE* + *(1 | Participant)*	6	39628.46	-19808	-	-
PoTJrDE2: PoTJ ~ *rDE* + *rDE:Session* + *(1 | Participant)*	7	39630.30	-19808	0.1609	0.6884
PoTJrDE3: PoTJ ~ *rDE* + *rDE:Direction* + *(1 | Participant)*	7	39277.47	-19632	352.83	<2e-16 ***
